# DSM-5 Attenuated Psychosis Syndrome in Adolescents Hospitalized With Non-psychotic Psychiatric Disorders

**DOI:** 10.3389/fpsyt.2020.568982

**Published:** 2020-10-21

**Authors:** Gonzalo Salazar de Pablo, Daniel Guinart, Barbara A. Cornblatt, Andrea M. Auther, Ricardo E. Carrión, Maren Carbon, Sara Jiménez-Fernández, Ditte L. Vernal, Susanne Walitza, Miriam Gerstenberg, Riccardo Saba, Nella Lo Cascio, Martina Brandizzi, Celso Arango, Carmen Moreno, Anna Van Meter, Paolo Fusar-Poli, Christoph U. Correll

**Affiliations:** ^1^Early Psychosis: Interventions and Clinical-Detection (EPIC) Lab, Department of Psychosis Studies, Institute of Psychiatry, Psychology & Neuroscience, King's College London, London, United Kingdom; ^2^Department of Child and Adolescent Psychiatry, Centro de Investigación Biomédica en Red de Salud Mental, General Universitario Gregorio Marañón School of Medicine, Institute of Psychiatry and Mental Health, Hospital Instituto de Investigación Sanitaria Gregorio Marañón (IiSGM), Universidad Complutense, Madrid, Spain; ^3^Department of Psychiatry, The Zucker Hillside Hospital, Northwell Health, Glen Oaks, NY, United States; ^4^Department of Psychiatry and Molecular Medicine, Donald and Barbara Zucker School of Medicine at Hofstra/Northwell, Hempstead, NY, United States; ^5^Institute for Behavioral Science, The Feinstein Institutes for Medical Research, Manhasset, NY, United States; ^6^Child and Adolescent Mental Health Unit, Jaén Medical Center, Jaén, Spain; ^7^Department of Psychiatry, University of Granada, Granada, Spain; ^8^Research Unit for Child- and Adolescent Psychiatry, Aalborg University Hospital, Aalborg, Denmark; ^9^Psychiatric University Hospital Zurich, Department of Child and Adolescent Psychiatry and Psychotherapy, Zurich, Switzerland; ^10^Department of Mental Health, Rome, Italy; ^11^Prevention and Early Intervention Service, Department of Mental Health, Rome, Italy; ^12^Local Health Agency Rome 1, Santo Spirito in Sassia Hospital, Department of Mental Health, Inpatient Psychiatric Unit, Rome, Italy; ^13^Department of Brain and Behavioral Sciences, University of Pavia, Pavia, Italy; ^14^Outreach and Support in South London Service, South London and Maudsley National Health Service Foundation Trust, London, United Kingdom; ^15^Department of Child and Adolescent Psychiatry, Charité Universitätsmedizin, Berlin, Germany

**Keywords:** Attenuated Psychosis Syndrome (APS), adolescence, epidemiology, risk, psychosis, prevention

## Abstract

**Introduction:** Although attenuated psychotic symptoms often occur for the first time during adolescence, studies focusing on adolescents are scarce. Attenuated psychotic symptoms form the criteria to identify individuals at increased clinical risk of developing psychosis. The study of individuals with these symptoms has led to the release of the DSM-5 diagnosis of Attenuated Psychosis Syndrome (APS) as a condition for further research. We aimed to characterize and compare hospitalized adolescents with DSM-5-APS diagnosis vs. hospitalized adolescents without a DSM-5-APS diagnosis.

**Methods:** Interviewing help-seeking, hospitalized adolescents (aged 12–18 years) and their caregivers independently with established research instruments, we (1) evaluated the presence of APS among non-psychotic adolescents, (2) characterized and compared APS and non-APS individuals regarding sociodemographic, illness and intervention characteristics, (3) correlated psychopathology with levels of functioning and severity of illness and (4) investigated the influence of individual clinical, functional and comorbidity variables on the likelihood of participants to be diagnosed with APS.

**Results:** Among 248 consecutively recruited adolescents (age=15.4 ± 1.5 years, females = 69.6%) with non-psychotic psychiatric disorders, 65 (26.2%) fulfilled APS criteria and 183 (73.8%) did not fulfill them. Adolescents with APS had higher number of psychiatric disorders than non-APS adolescents (3.5 vs. 2.4, *p* < 0.001; Cohen's d = 0.77), particularly, disruptive behavior disorders (Cramer's V = 0.16), personality disorder traits (Cramer's V = 0.26), anxiety disorders (Cramer's V = 0.15), and eating disorders (Cramer's V = 0.16). Adolescents with APS scored higher on positive (Cohen's d = 1.5), negative (Cohen's d = 0.55), disorganized (Cohen's d = 0.51), and general symptoms (Cohen's d = 0.84), and were more severely ill (Cohen's d = 1.0) and functionally impaired (Cohen's d = 0.31). Negative symptoms were associated with lower functional levels (Pearson ρ = −0.17 to −0.20; *p* = 0.014 to 0.031). Global illness severity was associated with higher positive, negative, and general symptoms (Pearson ρ = 0.22 to 0.46; *p* = 0.04 to *p* < 0.001). APS status was independently associated with perceptual abnormalities (OR = 2.0; 95% CI = 1.6–2.5, *p* < 0.001), number of psychiatric diagnoses (OR = 1.5; 95% CI = 1.2–2.0, *p* = 0.002), and impaired stress tolerance (OR = 1.4; 95% CI = 1.1–1.7, *p* = 0.002) (*r*^2^ = 0.315, *p* < 0.001).

**Conclusions:** A considerable number of adolescents hospitalized with non-psychotic psychiatric disorders meet DSM-5-APS criteria. These help-seeking adolescents have more comorbid disorders and more severe symptoms, functional impairment, and severity of illness than non-APS adolescents. Thus, they warrant high intensity clinical care.

## Introduction

Psychotic disorders, such as schizophrenia, are usually preceded by a clinical high-risk for psychosis (CHR-P) state (1), which is characterized by subtle symptoms, functional impairment and help-seeking behavior (2–4), as well as non-psychotic comorbidity (5, 6). The CHR-P state, which includes individuals at ultra-high risk for psychosis and/or those with basic symptoms, has allowed preventive efforts to be implemented (7, 8). This area of clinical research has grown until it has become one of the most established preventive approaches in psychiatry (7, 8).

The achievements and challenges of the CHR-P paradigm have been recently appraised by an umbrella review (9). In brief, three CHR-P subgroups have been established: attenuated psychotic symptoms; brief limited and intermittent psychotic symptoms (BLIPS) and genetic risk and deterioration (GRD) syndrome (9, 10). There are substantial diagnostic (11), prognostic (10, 12), clinical (13), and therapeutic (14) differences across these three subgroups. For example, psychosis risk is higher in the BLIPS group (38%) than in the attenuated psychotic symptoms group (24%) and higher in both groups than in the GRD group (8%) at >48 months follow-up (10).

**Figure 1:**
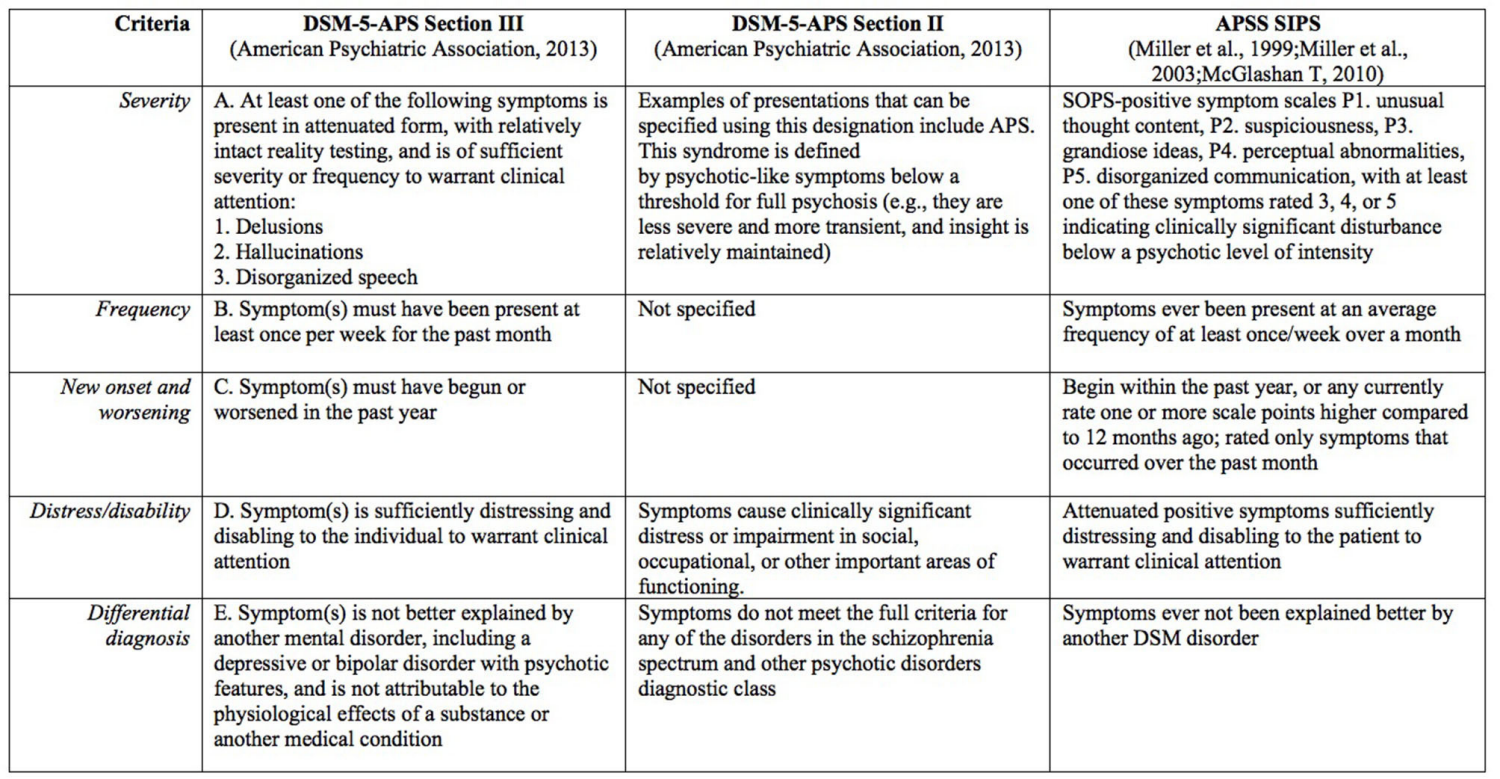
DSM-5-APS Attenuated Psychosis Syndrome diagnostic criteria compared with SIPS operationalization [adapted from (Gerstenberg et al. (22); Salazar De Pablo et al. (21)]. APS, Attenuated Psychosis Syndrome; APSS, Attenuated Positive Symptoms Syndrome; SIPS, Structured Interview for Psychosis-Risk Syndromes.

Although most research and clinical studies have evaluated the three groups together (15–17), the most common group by far is the attenuated psychotic symptoms group, which includes 85% of CHR-P individuals (10). Psychosis-risk syndromes, including attenuated psychotic symptoms, are usually characterized using semi-structured interviews as the Structured Interview for Psychosis-Risk Syndromes (SIPS) (18, 19) or the Comprehensive Assessment of At-Risk Mental States (CAARMS) (1), which have comparable prognostic accuracy (20). In the SIPS, the characterization used is Attenuated Positive Symptoms Syndrome (APSS). Seven years ago, the DSM-5 introduced the Attenuated Psychosis Syndrome (APS) diagnosis in the research appendix, listed in both section II and section III (21) (Figure 1). This diagnosis is defined by the presence of delusions, hallucinations, or disorganized speech in attenuated form, but with sufficient severity and frequency to warrant clinical attention (23, 24) (Figure 1). The diagnostic, prognostic, and therapeutic characteristics of this diagnosis have been recently appraised by a systematic review and meta-analysis (21). This review concluded that DSM-5-APS criteria have received substantial concurrent and prognostic validation, mostly driven by research in adult populations (21). A previous study looking at the agreement between CAARMS and DSM-5-APS criteria found that the agreement was only moderate (kappa 0.59) (25). Meanwhile, as findings from other studies point out (26, 27), SIPS and DSM criteria for APS are more similar (Figure 1).

While most reports to date on APS are based on cohorts that also include adults (25, 28–30), APS features often occur for the first time in adolescence (31, 32). Broadly speaking, studies that focus on DSM-5-APS in adolescents are scarce (21, 22), and there are few studies on APS in adolescents in clinical care and hospital settings.

To our knowledge, only a few efforts have been made (22, 33, 34) to characterize APS, excluding other ultra-high risk criteria, and advance knowledge specifically in children and adolescents, comparing them to other help-seeking individuals. Among them, 22 APS individuals were compared to other treatment-seeking individuals and healthy controls regarding clinical and cognitive features (34), finding that APS was associated with impaired neurocognition. Also, APS was associated with self-reported internalizing problems and thought problems in a study with 7 APS adolescents (33). One further study without a comparison group found that an older age of APS presentation in adolescents (comparing 9–14 years vs. 15–18 years) was associated with better social and role functioning and fewer depressive symptoms (35).

There is little evidence on how many help-seeking adolescents accessing inpatient care meet APS criteria. Our preliminary data from the Adolescent Mood Disorder and Psychosis Study (AMDPS) clinical study compared the first 21 APS and 68 non-APS adolescents who were recruited and found that APS was present in 23.6% of psychiatrically hospitalized adolescents, who suffered from a broad range of psychiatric symptoms and disorders (22).

Although specific knowledge for APS is limited, CHR-P individuals show impairments in work, educational and social functioning as well as poor quality of life (9, 36). Furthermore, psychopathology can adversely influence functioning (37). Negative symptoms have been associated with functioning, both daily (38), work related (39) and real-world functioning (40). Among CHR-P individuals, the severity of attenuated positive and negative symptoms has been associated with some outcomes [e.g., transition to psychosis (9, 21)] but not with others [e.g., cannabis use (9)]. Our preliminary results showed that poorer functioning in adolescents with APS was associated with more severe attenuated positive, negative, and general symptoms (22).

In the CHR-P field, the influence of sociodemographic and clinical variables on diagnostic and treatment outcomes has been widely studied, particularly regarding the transition to psychosis (41–45). Unusual thought content and suspiciousness have been found to predict conversion to psychosis along with decline in social functioning, lower verbal learning and memory performance (46). However, there is no convincing evidence of the association between any variable and the onset of psychotic disorders according to a meta-analysis, and only attenuated positive psychotic symptoms and global functioning show suggestive evidence (47). The influence of demographic and clinical variables on the presence of APS, particularly in adolescents, is even less known. In the first 89 individuals recruited into AMDPS, lowest GAF score in the past year, and social isolation were independently associated with APS (22).

The current study analyzes the final sample of this cohort of hospitalized adolescents to (1) assess how many non-psychotic, help-seeking adolescents accessing inpatient care meet APS criteria, (2) describe and compare both groups regarding sociodemographic, illness and intervention characteristics, (3) correlate attenuated positive, negative, general and disorganized symptoms with the level of functioning and severity of illness, and (4) investigate the influence of individual clinical, functional and comorbidity variables, selected empirically, on the likelihood of participants to be diagnosed with APS.

Based on prior literature, we hypothesized that (1) a significant number of adolescents with non-psychotic psychiatric disorders would fulfill APS criteria, (2) APS individuals would report significant comorbidity, clinical burden and functional impairment that would exceed those of non-APS individuals, (3) severity of negative symptoms would be significantly associated with the level of functioning and severity of illness, and (4) APS status would be associated with specific attenuated positive symptoms and other clinical variables.

## Materials and Methods

### Design and Setting

AMDPS was registered at ClinicalTrials.gov (NCT01383915).

Participants were recruited consecutively into AMDPS between September 2009 and July 2017 from the Adolescent Child and Adolescent Inpatient Unit of The Zucker Hillside Hospital, New York, USA (48, 49). AMDPS is an ongoing, prospective study that aims to assess predictors of the development of bipolar disorder and psychotic disorders in hospitalized adolescents. Analyses for this study are restricted to baseline data. The protocol was approved by the Institutional Review Board of the North Shore-Long Island Jewish Health System in accordance with the Helsinki Declaration of 1975 and the UNESCO Universal Declaration on human rights. Written informed consent was obtained from subjects aged 18 or the guardians/legal representatives of minors, obtaining written assent from the minors.

### Participants

Inclusion criteria for AMDPS study were: (1) age 12–18 years; (2) hospitalized at the adolescent inpatient unit of The Zucker Hillside Hospital, a self-standing psychiatric hospital; (3) admission chart diagnosis of any bipolar-spectrum disorder, cyclothymia, major depressive disorder, depressive disorder not otherwise specified (NOS), dysthymia or mood disorder NOS, schizophrenia, schizoaffective disorder, schizophreniform disorder or psychotic disorder NOS, re-evaluated by research interview, using the Structured Clinical Interview for DSM Disorders (SCID) (50), supplemented for missing pediatric diagnoses by the Schedule for Affective Disorders and Schizophrenia for School-Age Children-Present and Lifetime version (K-SADS-PL) (51); (4) subject and guardian/caregiver (if subject <18) willing and able to provide written, informed consent/assent. Exclusion criteria were: (1) an estimated premorbid IQ <70; (2) DSM-5 clinical criteria for autism spectrum disorders or pervasive developmental disorder and (3) history of any neurological or medical condition known to affect the brain.

**Figure 2:**
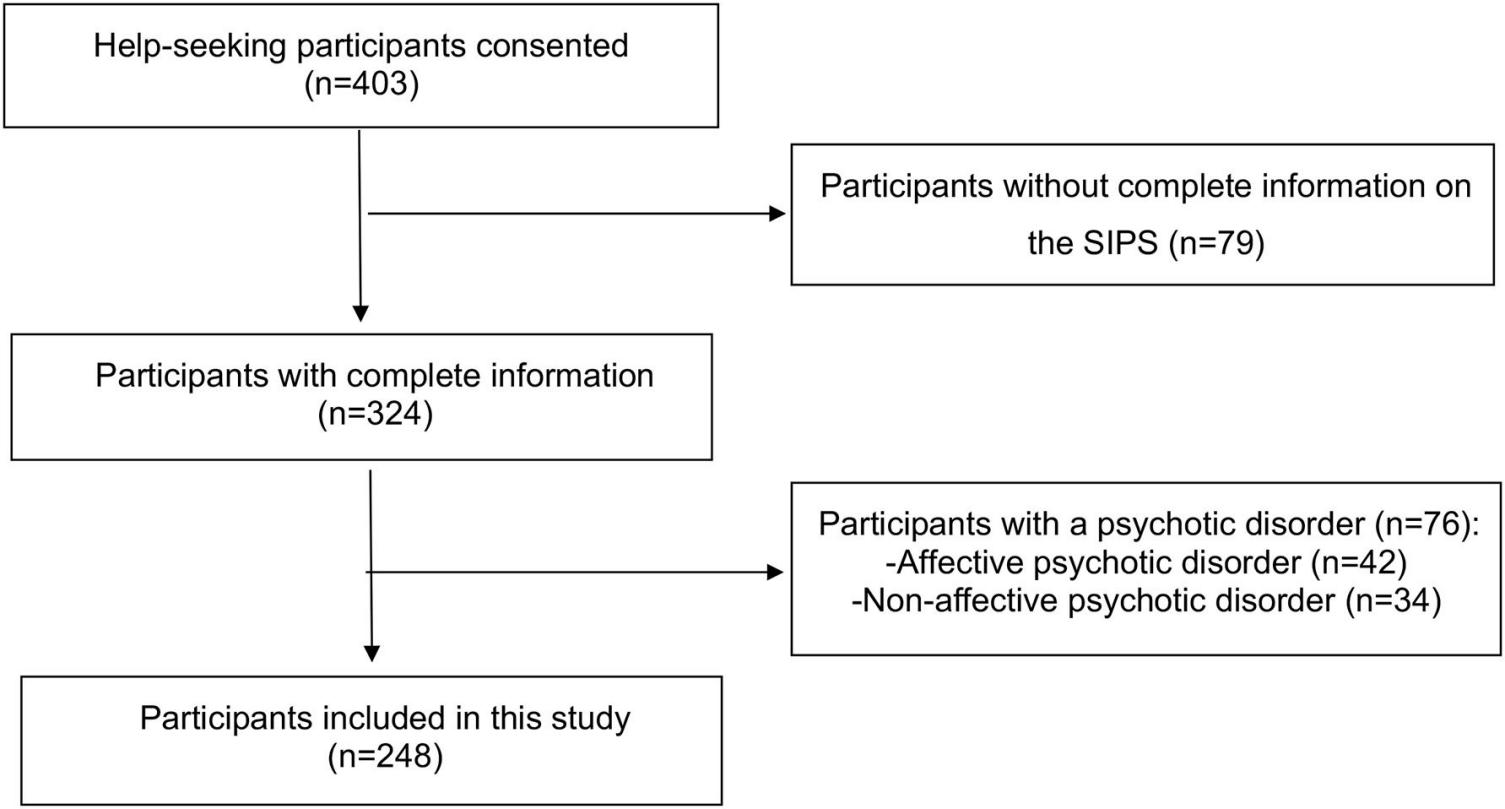
Flowchart outlining selection of study population. SIPS, Structured Interview for Psychosis-Risk Syndromes.

For the purpose of this study, we also excluded patients: (1) with a psychotic disorder according to DSM-5 criteria; (2) in whom the Structured Interview for Psychosis-Risk Syndromes, version 4.0 (52) was not completed (Figure 2).

Psychiatric diagnoses were established in diagnostic research consensus conferences based on in-person independent interview assessments of the adolescents and caregivers whenever possible. The interviews were typically conducted a few days after hospital admission. In consensus conferences, both assessments were integrated assuming that symptoms are more likely forgotten or hidden than invented or exaggerated. Also, SIPS items were discussed one by one for both interviews to reach to the correct value, and every psychiatric primary or comorbid diagnosis, including APS, was discussed among all the attendees and confirmed by the study lead (CUC). In order to conduct AMDPS assessments, experienced clinicians had to be certified by the study PI (CUC) after having gone through a structured training program, which involved observing several assessments, followed by conducting several assessments in front of one of the certified trainers, and presenting their ratings as part of a diagnostic consensus conference led by the study PI. All raters continually took part in the diagnostic consensus conference, during which all interview ratings were discussed and finalized as part of a group consensus, which served to assure validity of the ratings, facilitate interrater reliability via consensual rating, and avoid rater drift after completion of the initial training and certification.

### Diagnostic Assessments

The Structured Interview for Psychosis-Risk Syndromes (SIPS) (18, 19) is a semi-structured interview used to diagnose psychosis-risk syndromes in the last month. We used SIPS Version 4.0 (53). It includes four primary sections according to the symptoms evaluated: attenuated positive symptoms, negative symptoms, disorganized symptoms, and general symptoms. As part of the SIPS, the Scale of Prodromal Symptoms (SOPS) is used to determine whether participants meet research criteria for APSS. SIPS/SOPS psychometric instruments and DSM-5 criteria were both used to diagnose DSM-5-APS in a precise way.

### Clinical and Functional Assessments

Additional rating scales were administered to both adolescents and their caregivers, including the Clinical Global Impression–Severity scale (CGI-S; range = 1–7) to assess the overall severity of illness (54) and Global Assessment of Functioning (GAF) scale (55) to assess global functioning. Social and role functioning were assessed as well, using the Global Functioning: Social (GF: Social) and the Global Functioning: Role (GF: Role) (56, 57) scales. Insight was assessed using the Scale to Assess Unawareness of Mental Disorder (SUMD) (58), using three general awareness items: mental disorder, social consequences of mental disorder, and achieved effect of medication. Suicidality was assessed as the % of individuals who reported suicidal ideation lifetime and those with a history of at least one suicide attempt prior to admission.

### Data Analysis

We used descriptive statistics to characterize the study population, including diagnosis according to DSM-5 criteria, demographic variables, clinical characteristics and treatment characteristics. Between-group comparisons of categorical variables were performed using χ^2^-test or Fisher's exact test, whenever at least one cell contained ≤ 5 patients. For comparisons of continuous variables, we used *t*-test. The following effect sizes were calculated: (a) Cramer's V for χ^2^ (59), which was interpreted as follows: 0.1=small; 0.3=moderate; 0.5=large effect size; and (b) Cohen's d (60) for *t*-test, which was interpreted as follows: 0.2=small; 0.5=moderate; 0.8=large effect size, using effect size calculator for *t*-test (61). We correlated attenuated positive, negative, general and disorganized symptoms with the level of functioning and severity of illness using Pearson's correlation. We finally conducted a multivariable, backward logistic regression analysis, entering into the model variables that were significantly different (*p* < 0.05) between APS vs. non-APS groups in univariate analyses with data in >67% of subjects. For DSM-5 diagnoses, we entered into the multivariable model broad diagnostic categories (e.g., anxiety disorders), instead of single diagnoses (e.g., panic disorder), that were significantly different between the APS and non-APS group, in order to maximize power for the analyses. For the SIPS psychopathology symptoms, we included only individual items and not subscale sum scores to identify potentially clinically relevant symptoms that can guide clinical identification of APS status. The percent variance explained by the significant variables retained in the final multivariable logistic regression model was expressed as *r*^2^. Significance level was set at alpha=0.05, and all tests were two-tailed. Statistical analyses were performed with SPSS 21 for Windows software (IBM) (62).

## Results

### Demographic, Comorbidity and Treatment Characteristics

Altogether, 403 help-seeking adolescents and their guardians/legal representatives were consented into AMDPS. Of those, 79 (16.9%) were excluded from this study due to incomplete information on the SIPS, and of the remaining 324 patients, 76 (23.5%) had a psychotic disorder and were therefore also excluded. Finally, 248 hospitalized adolescents with non-psychotic psychiatric disorders were included in this study. Of those, 65 (26.2%) fulfilled DSM-5-APS criteria and 183 (83.8%) did not fulfill APS criteria (Figure 2). Agreement was 100% between DSM-5 clinical criteria and the SIPS.

Table 1 shows the demographic, illness and baseline treatment characteristics of the sample at the time of the interview. The average age of participants was 15.4 years (SD=1.5). Most participants were female (69.4%) and white (54.6%). There were no significant differences between the two groups in any of the demographic characteristics (Table 1).

**Table 1:** Demographic, comorbidity and treatment characteristics.

	**Total (*n* = 248)**	**APS (*n* = 65)**	**Non-APS (*n* = 183)**	***P-*value**	**Effect size**
Demographic characteristics					
Sex, male, *n* (%)	76 (30.6)	16 (24.6)	60 (32.8)	0.22	0.078
Age (years) mean ± SD	15.4 ± 1.5	15.5 ± 1.3	15.4 ± 1.5	0.63	0.070
Race/ethnicity, *n*, (%)^a^				0.60	0.11
White	124 (54.6)	32 (55.2)	92 (54.4)		
Black or African American	41 (18.1)	13 (22.4)	28 (16.6)		
Other	31 (13.7)	8 (13.8)	23 (13.6)		
Asian or Pacific Islander	28 (12.3)	5 (8.6)	23 (13.6)		
Indian American	3 (1.3)	0 (0.0)	3 (1.8)		
Estimated IQ, mean ± SD	108.4 ± 18.9	107.2 ± 17.8	108.8 ± 19.3	0.56	0.088
Lifetime consensus diagnoses, *n* (%)					
Number of psychiatric diagnoses	2.6 ± 1.5	3.5 ± 1.5	2.4 ± 1.4	**<0.001**	**0.77**
Depressive disorders	191 (77.0)	52 (80.0)	139 (76.0)	0.51	0.042
Major depressive disorder	137 (55.2)	42 (64.6)	95 (51.9)	0.077	0.11
Other specified depressive disorder	53 (21.4)	10 (15.4)	43 (23.5)	0.170	0.087
Persistent depressive disorder	18 (7.3)	5 (7.7)	13 (7.1)	0.87	0.010
Disruptive, impulse-control and conduct disorders	97 (39.1)	34 (52.3)	63 (34.4)	**0.011**	**0.16**
Attention-deficit/hyperactivity disorder	58 (23.4)	13 (20.0)	45 (24.6)	0.45	0.048
Oppositional defiant disorder	40 (16.1)	16 (24.6)	24 (13.1)	**0.03**	**0.14**
Conduct disorder	26 (10.5)	11 (16.9)	15 (8.2)	**0.049**	**0.12**
Disruptive behavior disorder not otherwise specified	11 (4.4)	4 (6.2)	7 (3.8)	0.43	0.050
Bipolar disorders	57 (23.0)	24 (36.9)	33 (18.0)	**0.002**	**0.20**
Other specified bipolar and related disorder	41 (16.5)	18 (27.7)	23 (12.6)	**0.005**	**0.18**
Bipolar I disorder	12 (4.8)	6 (9.2)	6 (3.3)	0.055	0.12
Bipolar II disorder	8 (3.2)	3 (4.6)	5 (2.7)	0.46	0.047
Personality disorder traits	48 (19.4)	24 (36.9)	24 (13.1)	**<0.001**	**0.26**
Borderline personality disorder traits	42 (16.9)	19 (29.2)	23 (12.6)	**0.002**	**0.20**
Other personality disorder traits	13 (5.2)	10 (15.4)	3 (1.6)	**<0.001**	**0.27**
Substance use disorders	39 (15.7)	13 (20.0)	26 (14.2)	0.27	0.070
Cannabis use disorder	31 (12.5)	9 (13.8)	22 (12.0)	0.70	0.024
Alcohol use disorder	14 (5.6)	6 (9.2)	8 (4.4)	0.14	0.093
Others	6 (2.4)	2 (3.1)	4 (2.2)	0.67	0.026
Trauma- and stressor-related disorders	38 (15.3)	8 (12.3)	30 (16.4)	0.43	0.050
Posttraumatic stress disorder	20 (8.1)	7 (10.8)	13 (7.1)	0.35	0.059
Adjustment disorder	19 (7.7)	2 (3.1)	17 (9.3)	0.11	0.10
Anxiety disorders	106 (42.7)	36 (55.4)	70 (38.3)	**0.016**	**0.15**
Panic disorder	63 (25.4)	23 (35.4)	40 (21.9)	**0.031**	**0.14**
Generalized anxiety disorder	37 (14.9)	16 (24.6)	21 (11.5)	**0.011**	**0.16**
Social phobia	24 (9.7)	10 (15.4)	14 (7.7)	0.07	0.11
Others	20 (8.1)	5 (7.7)	15 (8.2)	0.90	0.008
Obsessive-compulsive disorder	13 (5.2)	6 (9.2)	7 (3.8)	0.093	0.11
Specific phobia	9 (3.6)	6 (9.2)	3 (1.6)	**0.005**	**0.18**
Other diagnostic categories					
Eating disorders	20 (8.1)	10 (15.4)	10 (5.5)	**0.012**	**0.16**
Enuresis (not due to a general medical condition)	9 (3.6)	3 (4.6)	6 (3.3)	0.62	0.031
Treatment characteristics at time of the interview *n* (%)^b^					
Antipsychotics^c^	118 (53.6)	37 (66.1)	81 (49.4)	**0.031**	**0.15**
Antidepressants^d^	112 (50.9)	24 (42.9)	88 (53.7)	0.16	0.094
Mood stabilizers^e^	55 (25.0)	14 (25.0)	41 (25.0)	1.0	0.000
Lithium	41 (18.6)	9 (16.1)	32 (19.5)	0.57	0.038
Anxiolytics^f^	23 (10.5)	7 (12.5)	16 (9.8)	0.56	0.039
Others^h^	21 (9.5)	7 (12.5)	14 (8.5)	0.38	0.059
Antiepileptic drugs	18 (8.2)	6 (10.7)	12 (7.3)	0.42	0.054
ADHD medication^g^	4 (1.8)	0 (0.0)	4 (2.4)	0.24	0.080
Two or more drugs	91 (41.4)	22 (39.3)	69 (42.1)	0.71	0.025
Three or more drugs	25 (11.4)	7 (12.5)	18 (11.0)	0.76	0.021

*ADHD, Attention Deficit Hyperactivity Disorder; APS, Attenuated Psychosis Syndrome*.

a *Information available for 227 individuals*.

b *Information available for 220 individuals*.

c *Antipsychotics: aripiprazole, molindone, quetiapine, risperidone, lurasidone, ziprasidone, olanzapine, haloperidol, chlorpromazine, clozapine*.

d *Antidepressants: amitriptyline, nortriptyline, bupropion, citalopram, escitalopram, duloxetine, fluoxetine, paroxetine, sertraline, venlafaxine, mirtazapine*.

e *Mood stabilizers: lamotrigine, lithium, valproic acid*.

f *Anxiolytics/tranquilizers: clonazepam, lorazepam, hydroxyzine, buspirone*.

g *Anti-ADHD medications: atomoxetine, lisdexamphetamine, methylphenidate, modafinil, clonidine, guanfacine*.

h *Others: zolpidem, melatonin, propranolol, diphenhydramine, amlodipine*.

*Bold values indicate p <0.05 for between-groups analysis*.

APS individuals had a higher number of comorbid disorders (3.5 vs. 2.4, *p* < 0.001; Cohen's d = 0.77) compared to non-APS individuals. The most frequent in the total sample (APS plus non-APS) were depressive disorders (77.0%), particularly major depressive disorder (55.2%), followed by anxiety disorders (42.7%), and disruptive behavior disorders (39.1%). The following disorders were significantly more common in individuals with APS vs. non-APS: disruptive behavior disorders (*p* = 0.011; Cramer's V = 0.16), including oppositional defiant disorder (*p* = 0.03; Cramer's V = 0.14), and conduct disorder (*p* = 0.049; Cramer's V = 0.12); bipolar disorders (*p* = 0.002, Cramer's V = 0.20), including other specified bipolar and related disorders (*p* = 0.005; Cramer's V = 0.18)—also known as bipolar disorder NOS as defined by the COBY study criteria (63)–; personality disorder traits (*p* < 0.001; Cramer's V = 0.26), including borderline personality disorder traits (*p* = 0.002; Cramer's V = 0.20) and other personality disorder traits (*p* < 0.001; Cramer's V = 0.27); anxiety disorders (*p* = 0.016; Cramer's V = 0.15), including panic disorder (*p* = 0.031; Cramer's V = 0.14), generalized anxiety disorder (*p* = 0.011; Cramer's V = 0.16) and specific phobia (*p* = 0.005; Cramer's V = 0.18); and eating disorders (*p* = 0.012; Cramer's V = 0.16). The two groups did not differ in comorbid depressive disorders, substance use disorders, trauma and stressor-related disorders or enuresis (all *p* > 0.05).

Overall, the most used psychotropic medications at the time of the interview were antipsychotics (53.6%; *p* = 0.031), followed by antidepressants (50.9%; *p* = 0.16), and mood stabilizers (25.0%; *p* = 1.0). Antipsychotics, which were more common in the APS group (*p* = 0.031; Cramer's V = 0.15), were the only medication class that was significantly different between the groups. The use of multiple medications (use of two or more drugs or use of three or more drugs) was equally frequent in both groups (*p* = 0.71 to 0.76).

### Severity of Symptoms and Symptom Domains

Total attenuated positive (*p* < 0.001; Cohen's d = 1.5), negative (*p* < 0.001; Cohen's d = 0.55), disorganized (*p* < 0.001; Cohen's d = 0.51), and general (*p* < 0.001; Cohen's d = 0.84) symptom scores were significantly higher in APS individuals vs. non-APS hospitalized adolescents. All group-defining SIPS attenuated positive symptoms (unusual thought content, suspiciousness, grandiosity, perceptual abnormalities and disorganized communication) were significantly more severe in the APS group (Cohen's d = 0.39 to 1.3), with the largest effect size for perceptual abnormalities (Cohen's d = 1.3) (Table 2). Additionally, the following symptoms were more severe in the APS vs. non-APS group: social anhedonia (*p* < 0.001; Cohen's d = 0.57), avolition (*p* = 0.002; Cohen's d = 0.51), experiences of emotions and self (*p* < 0.001; Cohen's d = 0.54), bizarre thinking (*p* < 0.001; Cohen's d = 0.60), trouble with focus and attention (*p* = 0.001; Cohen's d = 0.53), sleep disturbances (*p* = 0.002; Cohen's d = 0.38), dysphoric mood (*p* = 0.004; Cohen's d = 0.34) and impaired stress tolerance (*p* < 0.001; Cohen's d = 0.63).

**Table 2:** Severity of structured interview of prodromal syndromes (SIPS) assessed symptoms and symptom domains.

	**Total (*n* = 248)**	**APS (*n* = 65)**	**Non-APS (*n* = 183)**	***P*-value**	**Effect size**
Structured interview of prodromal syndromes mean± SD					
Positive symptoms					
Total positive symptom score	3.2 ± 4.1	7.4 ± 4.6	1.9 ± 3.3	**<0.001**	**1.5**
Highest positive symptom score	1.8 ± 1.8	3.5 ± 1.3	1.2 ± 1.6	**<0.001**	**1.5**
P1 unusual thought content	0.73 ± 1.4	1.6 ± 1.6	0.41 ± 1.1	**<0.001**	**0.95**
P2 suspiciousness	0.84 ± 1.3	1.8 ± 1.6	0.48 ± 0.93	**<0.001**	**1.2**
P3 grandiosity	0.54 ± 1.2	0.89 ± 1.5	0.41 ± 1.1	**0.024**	**0.39**
P4 perceptual abnormalities/hallucinations	0.99 ± 1.7	2.3 ± 1.9	0.47 ± 1.2	**<0.001**	**1.3**
P5 disorganized communication	0.29 ± 0.86	0.63 ± 1.1	0.16 ± 0.68	**<0.001**	**0.58**
Negative symptoms					
Total negative symptom score	8.0 ± 6.22	10.4 ± 6.7	7.1 ± 5.8	**<0.001**	**0.55**
Highest negative symptom score	3.4 ± 1.83	3.8 ± 1.6	3.2 ± 1.9	**0.012**	**0.33**
N1 social anhedonia	1.5± 1.79	2.2 ± 1.9	1.2 ± 1.7	**<0.001**	**0.57**
N2 avolition	2.1 ± 2.0	2.9 ± 1.7	1.9 ± 2.0	**0.002**	**0.51**
N3 expression of emotions	0.88 ± 1.5	1.2 ± 1.6	0.75 ± 1.4	0.061	0.31
N4 experience of emotions and self	0.87 ± 1.7	1.6 ± 2.3	0.65 ± 1.4	**<0.001**	**0.54**
N5 ideational richness	0.20 ± 0.65	0.18 ± 0.18	0.21 ± 0.75	0.88	0.050
N6 occupational functioning	2.4 ± 2.1	2.5 ± 2.4	2.3 ± 2.0	0.31	0.11
Disorganized symptoms					
Total disorganized symptom score	3.1 ± 3.2	4.3 ± 3.7	2.7 ± 2.9	**<0.001**	**0.51**
Highest disorganized symptom score	2.2 ± 1.9	2.9 ± 1.7	2.03 ± 1.9	**0.003**	**0.47**
D1 odd behavior or appearance	0.16 ± 0.94	0.14 ± 1.4	0.17 ± 0.71	0.297	−0.03
D2 bizarre thinking	0.18 ± 0.7	0.48 ± 1.1	0.08 ± 0.4	**<0.001**	**0.60**
D3 trouble with focus and attention	1.9 ± 1.8	2.6 ± 1.7	1.66 ± 1.81	**0.001**	**0.53**
D4 impairment in personal hygiene	0.76 ± 1.7	0.86 ± 2.1	0.73 ± 1.54	0.45	0.08
General symptoms					
Total general symptom score	8.4 ± 4.5	11.0 ± 3.5	7.5 ± 4.4	**<0.001**	**0.84**
Highest general symptom score	4.3 ± 1.7	5.0 ± 1.1	4.1 ± 1.8	**<0.001**	**0.55**
G1 sleep disturbance	2.3 ± 1.9	2.8 ± 2.0	2.1 ± 1.8	**0.002**	**0.38**
G2 dysphoric mood	4.0 ± 2.1	4.5 ± 2.3	3.8 ± 2.0	**0.004**	**0.34**
G3 motor disturbance	0.14 ± 0.80	0.17 ± 1.4	0.13 ± 0.52	0.73	0.05
G4 impaired stress tolerance	1.9 ± 2.1	2.9 ± 2.3	1.6 ± 1.9	**<0.001**	**0.63**

*Bold values indicate p <0.05 for between-groups analysis*.

### Illness Severity, Functional Level, Illness Insight and Suicidality

Overall illness severity (CGI-S) was higher in the APS group (p <0.001) and the effect size was large (Cohen's d = 1.0). The mean current GAF score was 23.0 ± 11.9 in the APS group and 28.1 ± 17.9 in the non-APS group (*p* = 0.012; Cohen's d = 0.31). Scores for the highest functioning in the past year (*p* = 0.002; Cohen's d = 0.52) and lowest functioning in the past year (*p* = 0.002; Cohen's d = 0.38) were lower in the APS group as well (i.e., poorer functioning in the APS group). Unlike current role functioning, which did not differ significantly between the groups (*p* = 0.35), current social functioning was better in the non-APS group (*p* = 0.003; d = 0.66). Both groups did not differ regarding awareness of mental disorder or social consequences, suicidal ideation or suicidal attempts (all *p* > 0.05) (Table 3).

**Table 3:** Illness severity, functional level, illness insight and suicidality.

	**Total (*n* = 248)**	**APS (*n* = 65)**	**Non-APS (*n* = 183)**	***P*-value**	**Effect size**
Characteristics					
Illness severity: clinical global impressions-severity scale (CGI-S) mean ± SD^a^					
Overall severity of illness	4.2 ± 1.03	4.8 ± 0.94	3.9 ± 0.9	**<0.001**	**1.0**
Functional level: global assessment of functioning-scale (GAF) mean ± SD^b^					
Current GAF	26.8 ± 16.7	23.0 ± 11.9	28.1 ± 17.9	**0.012**	**0.31**
Highest GAF of past year	57.7 ± 14.7	52.2 ± 16.6	59.7 ± 13.5	**0.002**	**0.52**
Lowest GAF of past year	23.1 ± 15.0	18.9 ± 10.2	24.5 ± 16.0	**0.002**	**0.38**
Global functioning: role scale mean ± SD^c^					
Current role functioning	5.9 ± 1.8	5.7 ± 1.7	6.1 ± 1.9	0.35	0.20
Global functioning: social scale mean ± SD^c^					
Current social functioning	6.5 ± 1.7	5.8 ± 1.5	6.9 ± 1.7	**0.003**	**0.66**
Scale to assess unawareness of mental disorder mean ± SD^d^					
Awareness of mental disorder	2.2 ± 1.7	2.2 ± 1.6	2.2 ± 1.7	0.98	0.006
Awareness of the effect of medication	2.1 ± 1.5	2.2 ± 1.5	2.0 ± 1.5	0.45	0.14
Awareness of the social consequences	1.9 ± 1.5	1.9 ± 1.4	1.9 ± 1.5	0.99	0.0
Suicidality, *n* (%)^e^					
Suicidal ideation	131 (61.8)	38 (73.1)	93 (58.1)	0.29	0.067
Suicide attempts	21 (10.0)	8 (15.3)	13 (8.2)	0.19	0.082

a *Data available for 86 patients*.

b *Data available for 225 patients*.

c *Data available for 88 patients*.

d *Data available for 168 patients*.

e *Data available for 212 patients*.

*Bold values indicate p <0.05 for between-groups analysis*.

### Correlation Between Symptom Domains and Functioning (GAF)–Severity of Illness (CGI-S)

Total negative symptoms were significantly correlated with lower current functioning (Pearson ρ = −0.17; *p* = 0.031), lower lowest functioning in the past year (Pearson ρ= −0.20; *p* = 0.014) and lower highest functioning reached in the past year (Pearson ρ= −0.19; *p* = 0.022). Functioning was not significantly correlated with attenuated positive symptoms, disorganized symptoms or general symptoms. The severity of illness was associated with more severe SIPS positive, negative, disorganized and general symptoms (Pearson ρ = 0.22 to 0.46; *p* = 0.04 to *p* < 0.001) (Table 4).

**Table 4:** Correlation between Structured Interview of Prodromal Syndromes (SIPS) symptom domains and functioning as well as severity of illness.

	**Current GAF**		**Lowest GAF past year**		**Highest GAF past year**		**Severity of illness CGI-S**	
	**Pearson's Rho**	***p*-value**	**Pearson's Rho**	***p*-value**	**Pearson's Rho**	***p*-value**	**Pearson's Rho**	***p*-value**
Total SIPS positive symptom score	−0.034	0.66	−0.045	0.57	0.0005	0.95	0.46	**<0.001**
Total SIPS negative symptom score	−0.17	**0.031**	−0.20	**0.014**	−0.19	**0.022**	0.39	**<0.001**
Total SIPS disorganized symptom score	−0.04	0.58	−0.06	0.46	−0.043	0.61	0.22	**0.04**
Total SIPS general symptom score	0.095	0.21	0.082	0.3	0.017	0.36	0.45	**<0.001**

*CGI-S, Clinical Global Impression–Severity scale; GAF, Global Assessment of Functioning; SIPS, Structured Interview for Psychosis-Risk Syndromes*.

*Bold values indicate p <0.05 for between-groups analysis*.

### Multivariable Logistic Regression Analysis

Independent correlates of APS in the final model were perceptual abnormalities (OR = 2.0; 95% CI = 1.6–2.5, *p* < 0.001), number of psychiatric diagnoses (OR = 1.5; 95% CI = 1.2–2.0, *p* = 0.002), and impaired stress tolerance (OR = 1.4; 95%CI = 1.1–1.7, *p* = 0.002). The model including these three variables explained 31.5% of the variance (*r*^2^ = 0.315, *p* < 0.001) (Table 5).

**Table 5:** Results of the multivariable, backward elimination logistic regression analysis of variables distinguishing APS vs. non-APS at *p* < 0.05 in univariate analyses.

	**B**	**SE**	**Wald**	**Sig**	**OR**	**95.0% C.I**.	
						**Lower**	**Upper**
SIPS P4 perceptual abnormalities/hallucinations	0.69	0.11	38.9	**<0.001**	2.0	1.6	2.5
SIPS G4 impaired stress tolerance	0.31	0.10	9.8	**0.002**	1.4	1.1	1.7
Number of psychiatric diagnoses	0.42	0.14	9.5	**0.002**	1.5	1.2	2.0

*(r^2^ = 0.315, p <0.001)*.

*Bold values indicate p <0.05 for between-groups analysis*.

## Discussion

To our knowledge, this study is one of the very few and the largest to date to characterize and describe sociodemographic, illness and intervention characteristics in adolescents with APS vs. non-APS. Additionally, this study focused on help-seeking adolescents who had been admitted into an inpatient unit.

According to our results, 26.2% of the adolescents without a psychotic disorder diagnosis fulfilled APS criteria, a somewhat lower prevalence compared to a previous study including mostly adolescent outpatients (33%) (64, 65), but still a clinically significant and higher prevalence than the one found in non-help-seeking adolescents with disruptive behaviors (13%) (33). In the general population, a 7.2% meta-analytical prevalence of psychotic experiences was estimated in children and adults (66). In the Philadelphia Neurodevelopmental Cohort study, 15.5% of the 8–21 year old individuals reported significant psychotic symptoms and another 9.8% reported milder symptoms (67).

APS individuals had a higher number and distribution of comorbid conditions than non-APS individuals (Cohen's d = 0.77), particularly consisting of depressive disorders (5), anxiety disorders (5), and disruptive behavior disorders (68). This finding is clinically relevant because APS status has been associated with hospital treatment for mood and conduct disorders (33). Personality disorder traits, bipolar disorders, disruptive behavior disorders, eating disorders and anxiety disorders, were more frequent in the APS group than the non-APS group, although effect sizes were small. This result supports evidence of the association between APS (21, 22) as well as CHR-P (9, 69) with other comorbid mental disorders. Thus, comorbidity should not rule out APS, but, if anything, increase the diagnostic suspicion. On the other hand, it is also possible for APS status to be a byproduct of overlapping disease processes and expressions of non-psychotic disorders, lowering the true risk for developing a psychotic disorder in the future (22, 28, 70).

Regarding psychopharmacological treatment, as previously reported (22), a high percentage of our non-psychotic APS sample received atypical antipsychotics (66.1%), which was also high in the non-psychotic non-APS individuals (49.4%). This finding is worrying because no consistent meta-analytical evidence supports the use of atypical antipsychotic drugs in delaying or preventing transition to psychosis over other interventions (71, 72). However, it is also true that rates of antipsychotics were high in other diagnostic groups in this sample, including bipolar-spectrum disorders (49), which supports that atypical antipsychotic use is likely related to the reason for admission to the psychiatric unit and not only to efforts to treat attenuated psychotic symptoms or to prevent full-blown psychosis. Nevertheless, the widespread use of antipsychotics in adolescents for non-psychotic, predominantly depressive disorders is concerning due to the established adverse effects risks that atypical antipsychotics have in youth (73–77).

APS status was associated with a significantly higher severity of attenuated psychotic symptoms according to the SIPS. Effect sizes for these differences were moderate to large (Cohen's d = 0.51 to 1.5). Regarding individual items, differences were found in 13/19 items. Effect sizes were large for unusual thought content, suspiciousness and perceptual abnormalities (Cohen's d = 1.0 to 1.3), medium for disorganized communication, social anhedonia, avolition, experience of emotions and self, bizarre thinking, trouble with focus and attention and impaired stress tolerance (Cohen's d = 0.50 to 0.63), and small for grandiosity, sleep disturbances and dysphoric mood (Cohen's d = 0.34 to 0.39).

This greater severity in psychopathology also translated into greater illness severity (Cohen's d = 1.0) and poorer functioning (Cohen's d = 0.31 to 0.52), as found before (9, 22, 78, 79), including social functioning (Cohen's d = 0.66), but not role functioning. However, a previous study using the same instruments found that both social and role functioning were significantly more impaired in CHR-P individuals compared to controls from as early as age 12, which was our lower age limit (80). However, controls in that study were healthy, while in our sample, we compared hospitalized adolescents with vs. without APS who were likely admitted for symptoms related to other psychiatric disorders, which can explain the difficulties in role functioning as well as social and general functioning. The fact that all adolescents (APS and non-APS) reached stringent US criteria for inpatient care resulted in the low functioning scores found in both groups. Nevertheless, our results support previous evidence that APS status is associated with marked functional impairment (21, 81, 82). This finding is particularly relevant because functional impairment can be helpful to differentiate youth meeting CHR-P from other help-seeking individuals (83).

Interestingly, while illness severity was associated with overall psychopathology, including more severe SIPS total positive, negative, disorganized and general symptoms (Pearson ρ = −0.22 to −0.46), functioning (current, lowest and highest) was only and weakly (Pearson ρ = −0.17 to −0.20) correlated with total negative symptoms, but not with attenuated positive, disorganized and general symptoms. Negative symptoms have been associated with functioning (38–40), not only in schizophrenia, but also in other psychotic individuals, and non-psychotic depressed patients (84). This association was found to be greater with negative than attenuated positive symptoms (85), in line with our results. In contrast, trauma has been found to be correlated with the severity of attenuated positive symptoms but not with negative symptoms in CHR-P individuals (86); yet, CHR-P individuals' negative symptoms may impact the transition to psychosis even more than attenuated positive symptoms (87), although this has not been found consistently (53).

According to our results, perceptual abnormalities (OR=2.0), number of psychiatric diagnoses (OR=1.5), and impaired stress tolerance (OR=1.4) were independently associated with APS status. Among perceptual abnormalities, auditory perceptual abnormalities have been associated with a higher risk of psychosis, while visual perceptual abnormalities have been associated with a lower risk (88). While the number of psychiatric diagnoses was independently associated with APS status in our study, and while APS has previously been associated with comorbid mental disorders, the impact of the different comorbid conditions may vary (21, 22). The most common comorbid conditions in our sample, anxiety and depressive diagnoses, have been associated with impaired global functioning, as well as higher suicidality or self-harm behaviors, but not with transition to psychosis (5). Implications of the presence of other comorbid conditions in APS and their relevance for true risk for conversion to psychosis need further study, particularly in adolescents. Our results further support previous evidence that impaired stress tolerance is a core CHR-P feature, which is associated with more severe psychopathology (89). The presence of impaired stress tolerance has been also suggested to have therapeutic implications in CHR-P (90).

We also found that APS was associated with functioning in univariate analyses, but not in multivariable analyses, supporting that lower functioning is related to other features, including the presence and duration of attenuated positive symptoms (21, 91) and impaired stress tolerance (89). A model including disorganized communication, suspiciousness, verbal memory deficits, and decline in social functioning was found to predict conversion to psychosis (53). Due to having introduced the Global Functioning scales later into the study, they were only available in a subset of patients and could not be entered into the backward elimination logistic regression model. However, APS was associated with significantly lower levels of social functioning. Clinicians should thus monitor functioning, especially social functioning in adolescents with APS.

Finally, our results stress that in adolescent inpatients, DSM-5 APS is associated with higher severity of overall illness, lower functioning and impaired stress tolerance, requiring a higher intensity of clinical care compared to non-APS adolescents admitted into an inpatient unit. This result is supported by prior findings showing that youth with APS have complex medical histories and frequent comorbidities that require therapeutic attention (22, 28, 70). Research about effective treatments for DSM-5-APS has been limited (21), and evidence from studies analyzing CHR-P individuals—from which knowledge could arguably be applied to APS individuals—does not support one treatment over another (72). At the moment, at least needs-based interventions should be offered (9). Perceptual abnormalities and impaired stress tolerance may be targets of needs-based interventions in adolescents aiming to improve quality of life and aiming to reduce burden for them and their families. Still, prospective studies are needed to inform and develop guidelines regarding youth fulfilling APS criteria.

### Strengths and Limitations

The current study has several strengths and limitations that must be taken into consideration when interpreting its results. First, some symptom assessments were based on retrospective recall, which may be prone to recall bias. However, all SIPS symptoms were rated for presence in the last month. Second, the comparison group, including non-psychotic adolescents who fulfilled criteria for inpatient care in the US health care system, was otherwise heterogeneous and functionally impaired. The results should thus be interpreted in the context of help-seeking APS and non-APS samples in need of inpatient care. Third, data were not available to determine to what degree adolescents with APS sought help specifically for APS-related symptomology. Fourth, we did not collect some potentially relevant information, including the reason for the use of psychotropic medications or dosage, which could have relevant implications. Similarly, verbal memory deficits and other cognitive measures, which are relevant according to previous research, were not included in the current analysis. Fifth, we could not retrieve the data for the total number of patients fulfilling our inclusion criteria within our study timeframe outside of this study. Thus, we could not report the participation rate. Sixth, we did not test for interrater reliability of interviewers for all scales used in this study. However, using the same training, certification and ongoing recalibration system via mandatory presence and presentation of all rating scale scores for all interviewers as part of the regular diagnostic consensus conference (led by the study PI CUC) the interrater reliability of the BPSS-FP indices ranged from intraclass-correlations of 0.93–0.98 (92). Seventh, since the Clinical Global Impressions of Severity Scale and social and role function scales were introduced later into the study, data were not available in a sufficiently large number of patients to enter this variable into the multivariable regression analysis; Eighth, the final model obtained from the multivariable regression analysis was not validated, which may have led to overfitting, thus requiring replication and limiting its generalizability and consequently its implementation in clinical practice. Finally, the cross-sectional design precludes any analysis of the predictive value of APS.

Nevertheless, despite these limitations, the study has several strengths. First, this is the largest study to date to comprehensively describe and characterize DSM-5-APS in adolescents. Second, we used structured and validated assessments that were carried out independently and face-to-face for both adolescents and their parents or caregivers to obtain as precise information as possible. These assessments were led by experienced and internally certified Master or MD level clinicians and psychologists. Third, we focused on individuals with a wide variety of psychopathology and treatment characteristics, both in the DSM-5-APS group and in the non-APS comparison group, increasing clinical value vs. comparisons with healthy control subjects. Finally, focusing on APS individuals allowed us to obtain results from a more homogeneous high-risk sample.

## Conclusions

Approximately one in four adolescents hospitalized with non-psychotic disorders meet DSM-5-APS criteria. These help-seeking adolescents have more comorbid psychiatric disorders as well as more severe symptoms, functional impairment and global severity of illness. Thus, they warrant high intensity clinical care. To what degree APS in adolescents with existing and emerging non-psychotic mental disorders is predictive of future transition to a psychotic disorder and what the predictors are for such transition requires further prospective study.

## Data Availability Statement

Datasets generated for this study are included in the article. Additional data might be shared upon request from the first or corresponding author.

## Ethics Statement

The studies involving human participants were reviewed and approved by Institutional Review Board of the North Shore-Long Island Jewish Health System; Ethical Committee of Human Experimentation in the USA. Written informed consent to participate in this study was provided by the participants' legal guardian/next of kin.

## Author Contributions

GS had full access to all of the data in the study and takes responsibility for the integrity of the data and the accuracy of the data analysis. CC: study concept and design. GS, DG, BC, AA, RC, MC, SJ-F, DV, SW, MG, RS, NL, MB, and CC: acquisition of data. GS and CC: statistical analysis, drafting of the manuscript, administrative, technical, and material support. All authors critical revision of the manuscript for important intellectual content and interpretation of data.

## Conflict of Interest

DG has been a consultant for and/or has received speaker honoraria from Otsuka America and Janssen Pharmaceuticals. DV has received speaking fees from Lundbeck. SW has received in the last 5 years royalties from Thieme Hogrefe, Kohlhammer, Springer, Beltz. Outside professional activities and interests are declared under the link of the University of Zurich https://www.uzh.ch/prof/apps/interessenbindungen/client/. CA has been a consultant to or has received honoraria or grants from Acadia, Ambrosseti, Gedeon Richter, Janssen Cilag, Lundbeck, Otsuka, Roche, Sage, Servier, Shire, Schering Plow, Sumitomo Dainippon Pharma, Sunovion and Takeda. CM has acted as consultant or participated in DMC for Janssen, Servier, Lundbeck, Nuvelution, Angelini and Otsuka. PF-P has received grants and personal fees from Lundbeck and personal fees from Menarini. CC has been a consultant and/or advisor to or has received honoraria from: Acadia, Alkermes, Allergan, Angelini, Axsome, Gedeon Richter, Gerson Lehrman Group, IntraCellular Therapies, Janssen/J&J, LB Pharma, Lundbeck, MedAvante-ProPhase, Medscape, Neurocrine, Noven, Otsuka, Pfizer, Recordati, Rovi, Sumitomo Dainippon, Sunovion, Supernus, Takeda, and Teva. He has provided expert testimony for Bristol-Myers Squibb, Janssen, and Otsuka. He served on a Data Safety Monitoring Board for Lundbeck, Rovi, Supernus, and Teva. He received royalties from UpToDate and grant support from Janssen and Takeda. He is also a shareholder of LB Pharm. The remaining authors declare that the research was conducted in the absence of any commercial or financial relationships that could be construed as a potential conflict of interest.
